# Trajectories of stressful life events and long-term changes in mental health outcomes, moderated by family functioning? the TRAILS study

**DOI:** 10.1186/s13034-022-00544-0

**Published:** 2022-12-21

**Authors:** Lisette Wijbenga, Sijmen A. Reijneveld, Josue Almansa, Eliza L. Korevaar, Jacomijn Hofstra, Andrea F. de Winter

**Affiliations:** 1 Department of Health Sciences, University Medical Center Groningen, University of Groningen, Antonius Deusinglaan 1, FA10, 9713 AV Groningen, The Netherlands; 2 Research and Innovation Center for Rehabilitation, Hanze University of Applied Sciences, Groningen, The Netherlands

**Keywords:** Adolescence, Young adults, Stressful life events, Life course, Mental health, Family functioning

## Abstract

**Purpose:**

We assessed the association between trajectories of stressful life events (SLEs) throughout adolescence and changes in mental health from childhood to young adulthood. Further, we assessed whether family functioning moderated this association.

**Methods:**

Data of the first six waves of the TRAILS study (2001-2016; n = 2229) were used, a cohort followed from approximately age 11 to 23. We measured SLEs (death of a family member or other beloved one, delinquency, moving, victim of violence, parental divorce, and sexual harassment) at ages 14, 16 and 19. Family functioning was measured at all six time points using the Family Assessment Device (FAD), and mental health was measured through the Youth/Adult Self-Report at ages 11 and 23. Latent class growth analyses (LCGA) were used to examine longitudinal trajectories and associations.

**Results:**

We identified three SLE trajectories (low, middle, high) throughout adolescence, and found no significant associations between these trajectories and changes in mental health from childhood to young adulthood. Family functioning and SLE trajectories were significantly associated, however, the association of SLE trajectories and changes in mental health was not modified by family functioning. Mental health problems at age 11 increased the likelihood of high SLE trajectories during adolescence, and of experiencing negative family functioning.

**Conclusion:**

Experiencing SLEs throughout adolescence does not have a direct impact on changes in mental health from childhood to young adulthood, but early adolescence mental health problems increase the likelihood of experiencing SLEs.

**Supplementary Information:**

The online version contains supplementary material available at 10.1186/s13034-022-00544-0.

## Background

Adolescents experiencing stressful life events (SLEs) are at increased risk for mental health problems [1–3]. Exposure to stressful life events, such as moving, death of a family member, parental divorce, delinquency, violence or sexual harassment, can lead to serious long-term mental health issues [4]. A better understanding of associations and pathways over the life course, as well as possible moderators, is essential for the development of preventive interventions directed at adolescents experiencing stressful life events, and their families.

Many studies documented that chronic exposure to SLEs throughout adolescence can have a long-term detrimental impact on mental health [5–8]. For example, Bøe et al. [9] showed that adolescents who grow up in families with low socio-economic status and experience more frequent negative life events, had more mental health problems. Considering the potential long-term impact over a life course, SLEs throughout adolescence are seen as a serious public health issue. This is in line with the *accumulation of risk* hypothesis, stating that chronic exposure to a number of SLEs during adolescence may lead to health disadvantages over time [10]. Previous studies showed that a higher cumulative number of SLEs increased the likelihood of developing mental health problems [11–13]. Various studies suggested that the number of accumulated SLEs experienced at a point in time, as well as an increase or decrease of SLEs over time could affect young adults’ mental health [14, 15]. However, life-course studies with multiple time-points are scarce, especially those that cover trajectories of accumulated SLEs throughout the full adolescence and change in mental health in young adulthood. Therefore, the association between accumulation of SLEs throughout adolescence and changes in mental health from childhood to young adulthood, remains inadequately understood.

The family is crucial for the development of health and well-being of adolescents [16], therefore family functioning might serve as a moderator of the association between SLEs and change in mental health. Family functioning can be defined as a family’s organization, structure and transactional patterns, that ultimately affect all family members [17]. Positive family functioning refers to clear communication with few conflicts, cohesion within the family, and also good affect regulation [18]. Recent studies suggest that functioning within a family influences an adolescents’ response to certain adverse experiences [19]. Balistreri et al. [20] showed that adolescents who grew up in families with a more positive family functioning demonstrated higher levels of mental health, given exposure to SLEs. However, evidence lacks on the impact of exposure to SLEs throughout adolescence. Assessing trajectories of accumulated SLEs might provide a clearer insight in the possible impact of SLEs on changes in mental health throughout the life-course. This is needed even more, since no published studies have examined trajectories of stressful life events in adolescence, mental health in young adulthood, and family functioning measured from childhood until adulthood.

Therefore, the present paper aims to examine the association between trajectories of SLEs throughout adolescence and changes in mental health from childhood to young adulthood. Further, we assessed whether family functioning moderates this association.

## Methods

### Sample

We used data from the first six waves (T1-T6) of the Tracking Adolescents’ Individual Lives Survey (TRAILS). This is an ongoing prospective cohort study, which started with early adolescents living in five municipalities in the north of The Netherlands [21]. The sample contains both rural and urban areas and is representative for the northern part of The Netherlands. More information on the selection and follow-up of the TRAILS sample can be found elsewhere [22–24]. Participants in the first wave (T1, 2001–2002) included 2229 adolescents (response rate 76%; mean age = 11.1; SD = 0.6), followed by five waves, which took place every 2 or 3 years. Of the participants from T1, 96.4% participated again in the first follow-up (T2) until 72.6% in the final wave (T6) at age 26.

### Procedure

TRAILS collects data from parents and adolescents. Parents or caretakers (preferably the mother, 95.6%) were interviewed at their homes, on a wide range of topics, in the first wave (T1). At subsequent waves, they filled out various questionnaires. Adolescents filled out questionnaires at school or other testing locations under the supervision of TRAILS assistants. Written informed consent was obtained from all participants. Each of the six waves of the TRAILS study were approved by The Dutch Central Committee on Research involving Human Subjects. More details regarding the recruitment and assessment of the TRAILS study can be obtained elsewhere [21].

### Measures

#### Stressful life events (SLEs)

We assessed six SLEs at T2, T3, and T4 (age 14, 16, and 19, respectively). The six adolescent-reported SLEs included in this study were: death of a family member or other beloved one, delinquency (“In the past 2 years, have you been in contact with the police for doing something you are not allowed to do”), moving, victim of violence (“In the past 2 years, did someone used violence against you?”), and parental divorce (parents divorced or separated since the previous wave). Furthermore, we assessed sexual harassment using age-appropriate questions per wave. At T2, this regarded asking “In the past 2 years, did someone make sexual comments, jokes or movements towards you?”. At T3, this regarded sexual insinuations, and at T4 if the participant was sexually assaulted or raped. For all items, at T2 and T4, participants indicated in a questionnaire whether the life event had occurred in the last 2 years (resp. between T1 and T2; between T3 and T4) through a yes/no format. When adolescents reported one or more SLEs as present, we considered the missing values in the other events as no event (0). When all SLEs were marked as missing values, we considered this as non-response or a drop-out. At T3 participants responded to the same questions in interviews, using an Event History Calendar (EHC), a method to collect data through retrospectively reporting on life events [25]. Since this data collection was done via in-depth face-to-face interviews, it is reasonable to assume that no-response equals no-event. We created a sum variable of all SLEs per wave.

#### Family functioning

We measured family functioning with the parent-reported McMaster Family Assessment Device (FAD) [26]. This contained 12 statements, such as “In our family we express feelings to each other”, “We don’t get along well in our family”, or “We can count on each other’s support in difficult times”. Each statement consisted of four answer categories (e.g., “strongly agree”, “agree”, “disagree”, and “strongly disagree”). Our moderator score is a constructed mean variable of family functioning from wave 1 until wave 6, with a maximum score of 3.75. A higher score indicates a more negative family functioning.

#### Mental health

Mental health was assessed at T1 using the Youth Self Report (YSR), and at T5 and T6 using the Adult Self Report (ASR). The YSR and the ASR are both highly valid and reliable measurements [27, 28]. Adolescents answered 112 items on behavioral and emotional problems on a three-point scale (not true, somewhat or sometimes true, very true or often true), with a timeframe of 6 months. Externalizing problems (delinquent, aggressive and intrusive behavior), internalizing problems (physical complaints, withdrawn/depressed, and anxious/depressed) and other mental problems (attention, and thinking problems) were assessed and combined to a total problem score. Age-standardized scores of all scales combined were used, at a continuous-level. Our outcome score was the constructed mean score on young adults’ mental health problems at waves 5 and 6, i.e. young adulthood, with a maximum score of 1.23. Higher scores indicate more mental health problems. We considered scores above the 93th percentile to be in the borderline and clinical range of mental health problems (Achenbach and Rescorla, 2003).

#### Background characteristics

Participants provided data on age, sex, ethnicity, and SES, at baseline. SES entailed occupation and education of both mother and father, and family income combined.

#### Data handling and statistical analyses

First, descriptive statistics were calculated for all variables. The missing data was limited to a small number of observed SLEs (see Additional file 3: Table S1). Second, we identified developmental trajectories of SLEs over time (T2-T4) using latent class growth analyses (LCGA) [29], and assessed whether these were associated with mental health in childhood (T1), young adulthood (average of T5 and T6), and with changes in mental health (difference between T1 and T5/T6), by estimating their means across SLE trajectories. The number of SLEs at each timepoint was modelled using a Poisson distribution, and time was treated as categorical, so no trend shape was pre-defined. The trajectories were estimated via maximum-likelihood with robust standard errors and 1000 starting values, and missing values were assumed to be missing at random. The Bayesian Information Criterion (BIC), the Akaike Information Criterion (AIC), and the Lo-Mendell-Rubin Likelihood Ratio Test (LRT) were used to determine the most representative number of trajectories. Third, we assessed whether the relationship between SLE trajectories and changes in mental health were modified by family functioning (centered T1-T6) after adjusting for sex, ethnicity, and family SES. We also examined how SLE trajectories and mental health were related to family functioning. The LCGA were performed in Mplus version 8 [30], the descriptive analyses were conducted using SPSS v.25.0 software [31].

## Results

### Sample characteristics

Table 1 shows descriptive statistics of predictor and outcome variables. The sample consisted of 2229 adolescents at baseline (average age 11). Most adolescents were Dutch (87.0%), and around half of the sample was female (51.4%). 522 adolescents (24.4% of the sample) were raised in families with low socio-economic status. We also observe in Table 1 relatively higher average mental health problems at age 11 (0.34) compared to the observed value at age 23 (0.29).

**Table 1:** Descriptive statistics of the sample (N = 2229)

	T1	T2	T3	T4	T5/T6
Age in years, mean (SD)	11.11 (0.56)	13.57 (0.53)	16.28 (0.71)	19.08 (0.60)	23.08 (0.76)
Stressful life events, N (%)					
Death of a loved one		803 (37.4)	810 (42.2)	369 (19.6)	
Moving		316 (14.7)	387 (20.2)	516 (27.4)	
Parental divorce		89 (4.1)	80 (4.2)	77 (4.1)	
Delinquency		238 (11.1)	277 (14.4)	95 (5.0)	
Victim of violence		151 (7.0)	40 (2.1)	67 (3.6)	
Victim of sexual harassment		246 (11.4)	106 (5.5)	17 (0.9)	
Total, mean (SD)		0.9 (0.9)	0.9 (0.9)	0.6 (0.7)	
Family functioning, mean (SD)	1.77 (0.36)	1.64 (0.40)	1.65 (0.40)	1.68 (0.40)	1.65 (0.41)/1.71 (0.40)
Mental health^a^, mean (SD) [95% CI]	0.34 (0.19) [0.33; 0.35]				0.29 (0.20) [0.28; 0.30]

^a^YSR, ASR; standardized total problem score

### Associations between SLE trajectories and changes in mental health

**Fig. 1:**
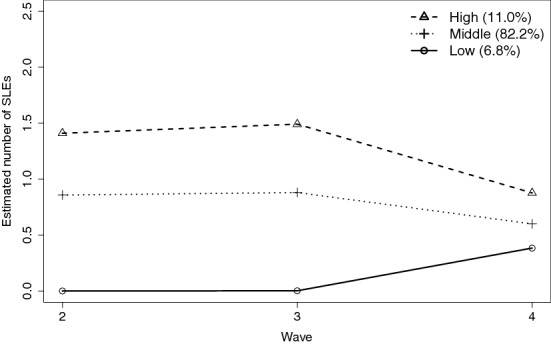
Trajectories of individuals categorized according by number of SLEs over time (T2-T4)

We identified three developmental trajectories of SLEs (low, middle, high; Table 2), and examined its association with changes in mental health from childhood to young adulthood. According to Nagin [32], the entropy was considered medium. Although AIC and BIC pointed towards a two-class solution, the Lo-Mendell-Rubin LRT Test assessed a significant difference in the three-class solution, which indicates that the model with three latent classes provided a better fit to the data than the two-class solution. The four-class solution resulted in an empty class. In line with the guidelines as provided by Schoot et al. [33], we decided on a 3-class model, both grounded on our theoretical expectations and the interpretability of our results (see Additional file 1: Figure S1, Additional file 2: Figure S1). Figure 1 shows a small low trajectory (6.8% of participants), a small high trajectory (11.0% of participants), and a relatively large middle trajectory containing most adolescents (82.2% of participants). Trajectories were based on the estimated number of SLEs, so not on different types of SLEs. Nevertheless, they did not differ regarding the relative shares of the constituting types of SLEs (see Additional file 3: Table S2). Trajectories seem to be converging over time with levels of SLEs coming closer together at T4.

**Table 2:** Comparative goodness of fit indices for the trajectory models of the number of SLEs at ages 13, 16, and 19

Number of classes	Akaike information criteria*	Bayes information criterion*	Lo-mendell-rubin LRT Test*	Entropy
1	13767.38	13784.41	–	
2	13726.54	13766.27	.00	0.53
**3**	**13731.48**	**13793.92**	**.04**	**0.59**

^*^The used model is indicated in bold

The results on the association between SLE trajectories and changes in mental health are presented in Table 3. No significant differences were found across the high (mean_change_ = − 0.07), middle (mean_change_ = − 0.01), and low (mean_change_ = − 0.06) trajectories of SLEs and changes in mental health over time (overall *p* = 0.334). However, we did find an association of trajectories of SLEs with mental health at baseline (T1) and with mental health at age 23 (T5/T6). Levels of mental health problems were higher for high SLE trajectories (mean_T1_ = 0.67 and mean_T5/T6_ = 0.65) than for low SLE trajectories (mean_T1_ = 0.18 and mean_T5/T6_ = 0.10), and middle SLE trajectories (mean_T1_ = 0.31 and mean_T5/T6_ = 0.25; overall *p* = 0.000).

**Table 3:** Associations between membership of SLE trajectory with changes in mental health^a^ at age 23, and the moderating effect of Family Functioning (expected means, estimates and confidence intervals across trajectories)

*Descriptives per trajectory*	Low SLE trajectory^b^		Middle SLE trajectory^b^		High SLE trajectory^b^		*p-* value^f^
	Mean	95%CI	Mean	95%CI	Mean	95%CI	
Mental health_T1_^a^	0.18	[0.15; 0.20]	0.31	[0.30; 0.32]	0.67	[0.64; 0.70]	0.000
Mental health_T5/T6_^a^	0.10	[0.83; 0.12]	0.25	[0.24; 0.26]	0.65	[0.61; 0.68]	0.000
Family Functioning	1.65	[1.54; 1.77]	1.67	[1.65; 1.69]	1.98	[1.94; 2.02]	0.000
Mental health_change_^acd^	− 0.07	[− 0.11; − 0,04]	− 0.06	[− 0.07; − 0.05]	− 0.01	[− 0.09; 0.08]	0.334
*Moderator model*^*cde*^	B	95%CI	B	95%CI	B	95%CI	*p-* value^f^
Intercept	− 0.09	[− 0.15; − 0.04]	− 0.06	[− 0.07; − 0.04]	− 0.03	[− 0.13; 0.06]	0.324
Family Functioning	− 0.12	[− 0.32; 0.09]	0.04	[− 0.02; 0.10]	0.05	[− 0.23; 0.31]	0.383

Estimates (B) with Standard Errors (SE)

^a^Mental health is measured using standardized YSR/ASR scores

^b^More information on the trajectories can be found in Table 2 and Fig. 1

^c^Estimated means of the difference between mental health T5/T6 and mental health T1

^d^Controlled for the variables sex, socioeconomic status, and ethnicity

^e^This model demonstrates coefficients for the association of Family Functioning with changes in mental health across trajectories of SLEs

^f^Test of differences of means across trajectory classes

### Associations of family functioning, SLE trajectories, and changes in mental health

Next, we assessed how SLE trajectories and changes in mental health were related to family functioning. We found that family functioning was poorer in high SLE trajectories compared to the other classes (*p* < 0.001; see Table 3). However, within SLE trajectories, family functioning did not modify the effect of the SLE trajectories on changes in mental health from childhood to young adulthood (*p* = 0.383).

## Discussion

The present paper is the first to examine the association between trajectories of SLEs throughout adolescence and changes in mental health from childhood to young adulthood, and how these are related to family functioning. We identified three SLE trajectories (low, middle, high) throughout adolescence, and found no association between these trajectories and changes in mental health from childhood to young adulthood. However, mental health problems and negative family functioning occurred more in high SLE trajectories than in middle and low SLE trajectories. Family functioning did not modify the association of SLE trajectories and changes in mental health.

We found that changes in mental health from childhood to young adulthood could not be explained by experiencing SLEs throughout adolescence. This is in contrast to other studies that demonstrated an association between experiencing SLEs and mental health [34]. However, these studies were either cross-sectional or only used a few time-points. To the best of our knowledge, no study focused specifically on the changes in mental health over time. Our findings could be interpreted in the light of resilience. Since resilience strongly associates with mental health [35], it may be that the levels of SLEs are relatively similar throughout childhood and adolescence, but there is a lower resilience for such events throughout adolescence compared to childhood [36]. This in turn, may correspond with our finding that, within the different SLE trajectories, mental health problems at age 11 seem to be an important predictor of mental health problems in young adulthood.

Our results show that adolescents with more mental health problems (age 11 and age 23) experienced more SLEs throughout adolescence, while adolescents with less mental health problems also experienced less SLEs. Furthermore, trajectories seem to converge in late adolescence, which might be explained by the Dutch school system. While most adolescents transition out of secondary education, this often pairs with moving out of the parental home and an enlargement of their social contexts. A possible explanation for our findings may also be that the impact of SLEs shows a larger variation in combination with other constructs, such as personality or support by family or friends. For example, the complexity of the association might come from the SLEs themselves. Weinberg et al. [37] demonstrated the importance of the interrelationship of the objective type of a SLE, the subjective reaction to that SLE, and the adolescent’s personality characteristics. Individuals who experienced the SLEs as a threat and scored high on the personality trait neuroticism were more at risk of mental health problems. While a similar study showed that extraversion might serve as a protective factor between experiencing SLEs and developing mental health problems [38]. Prior research demonstrated that an accumulation of SLEs had different effects on long-term health outcomes [39], with not only devastating effects, but also improvement after decline, and even an increase in well-being later on. Thus, not solely the SLEs, but also their interaction with, for example, personality and other possible factors may affect outcomes later in life.

In our study, family functioning (age 11 to 26) and SLE trajectories were associated, i.e., adolescents with more SLEs throughout adolescence grew up in families with more negative family functioning. Consistent with previous research [40], family functioning did not add additional risk or act as a protective factor on the relation between SLE trajectories throughout adolescence and changes in mental health in young adulthood. Our study transcended recent research demonstrating that negative family functioning predicted mental health problems [41, 42]. An explanation may be that we examined family functioning across SLE trajectories within a general community population in a longitudinal design with multiple time points, while most studies aimed at specific high-risk groups in a cross-sectional design. Besides, to the best of our knowledge, no previous study focused on trajectories of SLEs, family functioning, and specifically changes in mental health over the life course, whereas we did throughout full adolescence. Another explanation for our findings might be that, throughout adolescence, the effect of the family becomes more restricted in comparison to the influences of the social context, such as peers [43].

### Strengths and limitations

This life course study has several strengths. First, it covered frequent assessments throughout 15 years in a large population-based cohort, including follow-up waves with high response rates. Second, we described the trajectories based on a large range of SLEs. Third, we assessed mental health before and after the SLEs, which allowed to control for mental health problems before the SLE and to assess mental health impact in the longer term, in this way passing some of the challenges of previous studies [44, 45]. However, also some limitations should be considered**.** First**,** we examined family functioning reported from the perspective of the parents. Adolescents might have a different perception of the family [46, 47]. However, a recent study deemed the family functioning instrument (McMasters FAD) well-suited to measure family dynamics, using either the adolescents’ or the caregivers’ view, across time [48]. Second, consistent with other studies [49], family functioning was a rather stable construct over time with adolescents mainly experiencing positive family functioning. This limited the power to detect moderation. In our sample, family functioning was homogeneous within trajectories, although different across trajectories, i.e., adolescents living in families with lower levels of family functioning belonged to a higher SLE trajectory. Thus, even though our cohort still included both adolescents that experienced negative and positive family functioning, our findings should be confirmed in more diverse adolescent samples. Furthermore, our study focused on trajectories based on number of SLEs measured throughout adolescence. Therefore, we did not include the level or potential differences in severity of the SLEs, which might have different associations with mental health [8]. From a developmental perspective, SLEs can act as risk factors for negative mental health outcomes, which could mean that experiencing more SLEs exceeds the adverse effects of single SLEs on mental health [50].

### Implications

Our results suggest that adolescents are more likely to experience many SLEs throughout adolescence if they have more mental health problems at age 11, indicating that these young adolescents deserve more attention. On top, our results also highlight the importance of further research on these complex associations throughout childhood and during adolescence. For example, the actual impact of high levels of SLEs during childhood may be underlying both the higher SLE levels during adolescence and the higher level of mental health problems at age 11. It deserves further study to which extent this association is influenced by other background factors, such as the school context, peer support or the impact of the neighbourhood. Further research is also required to untangle why number of SLEs decrease over time in those adolescents with higher numbers of SLEs. Furthermore, in line with emerging literature, such as March‑Llanes [51], we suggest that future studies should not only focus on the number of SLEs, but also whether the type of SLEs might matter for long-term mental health.

## Conclusion

In conclusion, this study shows the importance of life-course research on trajectories of SLEs and their long-term associations with mental health. Our findings suggest that experiencing SLEs throughout adolescence does not have a direct effect on long-term mental health. However, adolescents’ mental health at age 11 predicts higher levels of SLEs during adolescence. Finally, adolescents that grow up in a family with negative quality of functioning seemed to experience more SLEs throughout adolescence.

## Supplementary Information

**Additional file 1: Figure. S1. Trajectories of individuals for one-class solution categorized according by number of SLEs over time (T2-T4)**

**Additional file 2: Figure. S2. Trajectories of individuals for two-class solution categorized according by number of SLEs over time (T2-T4)**

**Additional file 3: Table S1.** Missings per type of SLEs and wave; (n, %; N= 2229). **Table S2.** Descriptive statistics of the percentage* of SLEs per trajectory, type of SLE and wave (N=2,229)

## Data Availability

The TRAILS data are accessible to researchers outside the TRAILS consortium, via DANS EASY. Data are available free of charge, with the exception of 2500 euros as a contribution to the TRAILS infrastructure. Access to the data can be obtained by submitting a publication proposal (see www.trails.nl for more information).

## Code availability

Codes can be requested from the TRAILS consortium (see www.trails.nl for more information).
