# Supplementary material for: Trajectories of stressful life events and long-term changes in mental health outcomes, moderated by family functioning? the TRAILS study
Source: Child Adolesc Psychiatry Ment Health. 2022 Dec 21;16:106. doi: 10.1186/s13034-022-00544-0 (PMC9768997; doi:10.1186/s13034-022-00544-0)
Supplement: Supplementary file 3 — Additional file 3: Table S1. Missings per type of SLEs and wave; (n, %; N= 2229). Table S2. Descriptive statistics of the percentage* of SLEs per trajectory, type of SLE and wave (N=2,229) [file 13034_2022_544_MOESM3_ESM.docx]

# **Additional file**

| **Table S1.** Missings per type of SLEs and wave; (n, %; N= 2,229) | | | | | | |
| --- | --- | --- | --- | --- | --- | --- |
| Observations included | Death of a loved one | Parental divorce | Delinquency | Victim of violence | Moving | Victim of sexual harassment |
| T2 (N=2,149) | 63 (2.9) | 7 (0.3) | 62 (2.9) | 61 (2.8) | 63 (2.9) | 63 (2.9) |
| T3* (N=1,816) | * | * | * | * | * | * |
| T4 (N=1,881) | 227 (12.1) | 1 (0.1) | 180 (9.6) | 178 (9.5) | 211 (11.2) | 181 (10.0) |
| * At T3 participants responded to the same questions as T2 and T4 through interviews. Their responses were coded as no-response equaling no-event. | | | | | | |

| **Table S2.** Descriptive statistics of the percentage* of SLEs per trajectory, type of SLE and wave (N=2,229) | | | | | | |
| --- | --- | --- | --- | --- | --- | --- |
|  | Death of a loved one | Parental divorce | Delinquency | Victim of violence | Moving | Victim of sexual harassment |
| Low SLE trajectory, % |  |  |  |  |  |  |
| T2 | 0.1% | 0.0% | 0.0% | 0.0% | 0.0% | 0.0% |
| T3 | 0.2% | 0.0% | 0.0% | 0.0% | 0.0% | 0.0% |
| T4 | 13.9% | 2.0% | 2.7% | 1.0% | 15.1% | 0.3% |
| Middle SLE trajectory, % |  |  |  |  |  |  |
| T2 | 38.2% | 4.0% | 10.8% | 6.8% | 14.3% | 11.3% |
| T3 | 38.6% | 3.7% | 12.3% | 1.6% | 17.5% | 4.6% |
| T4 | 17.3% | 3.5% | 4.3% | 2.9% | 23.7% | 0.7% |
| High SLE trajectory, % |  |  |  |  |  |  |
| T2 | 53.7% | 7.4% | 19.7% | 13.2% | 26.2% | 20.1% |
| T3 | 53.0% | 6.5% | 25.0% | 5.1% | 32.5% | 10.1% |
| T4 | 23.6% | 4.9% | 6.5% | 5.7% | 33.2% | 1.6% |
| * Percentages were estimated based on the exact class membership posterior probability. | | | | | | |
